# Novel Indexes in the Assessment of Cardiac Enlargement Using Chest Radiography: A New Look at an Old Problem

**DOI:** 10.3390/jcm14030942

**Published:** 2025-02-01

**Authors:** Patrycja S. Matusik, Tadeusz J. Popiela, Paweł T. Matusik

**Affiliations:** 1 Department of Diagnostic Imaging, University Hospital, 30-688 Kraków, Poland; patrycja.s.matusik@gmail.com (P.S.M.); msjpopie@cyf-kr.edu.pl (T.J.P.); 2 Chair of Radiology, Jagiellonian University Medical College, 31-008 Kraków, Poland; 3 Department of Electrocardiology, Institute of Cardiology, Faculty of Medicine, Jagiellonian University Medical College, 31-008 Kraków, Poland; 4 Department of Electrocardiology, St. John Paul II Hospital, 31-202 Kraków, Poland

**Keywords:** cardiomegaly, left ventricular hypertrophy, left ventricular dilatation, cardio–thoracic ratio, chest radiograph, cardiac magnetic resonance, transverse cardiac diameter

## Abstract

**Background:** Chest X-rays are among the most frequently used imaging tests in medical practice. We aimed to assess the prognostic value of the cardio–thoracic ratio (CTR) and transverse cardiac diameter (TCD) and compare them with novel chest X-ray parameters used in screening for cardiac enlargement. **Methods:** CTR, TCD, and five other non-standard new radiographic indexes, including basic spherical index (BSI), assessing changes in cardiac silhouette in chest radiographs in posterior–anterior projection were related to increased left ventricular end-diastolic volume (LVEDV) and left ventricular hypertrophy (LVH) assessed in cardiac magnetic resonance imaging (CMR). **Results:** TCD, CTR, and BSI were the best predictors of both LVH and increased LVEDV diagnosed in CMR. The best sensitivity, along with good specificity in LVH prediction, defined as left ventricular mass/body surface area (BSA) > 72 g/m^2^ in men or >55 g/m^2^ in women, was observed when TCD and BSI parameters were used jointly (69.2%, 95% confidence interval [CI]: 52.4–83.0% and 80.0%, 95% CI: 51.9–95.7%, respectively). In the prediction of cardiac enlargement defined as LVEDV/BSA > 117 mL/m^2^ in men or >101 mL/m^2^ in women, BSI > 137.5 had the best sensitivity and specificity (85.0%, 95% CI: 62.1–96.8% and 82.4%, 95% CI: 65.5–93.2%, respectively). **Conclusions:** TCD may be valuable in the assessment of patients suspected of having cardiac enlargement. CTR and BSI serve as complementary tools for a more precise approach. TCD appears particularly useful for the prediction of LVH, while BSI demonstrates greater utility as an indicator of increased LVEDV.

## 1. Introduction

Chest radiographs are one of the most commonly performed radiological investigations in clinical practice [1] and are widely used in screening for cardiac enlargement. However, quantitative aspects of cardiac enlargement based on chest radiographs are still under question and the value of chest radiography in the assessment of left ventricular (LV) size is controversial [1]. In 1919, Danzer [2] described the most common method of cardiac enlargement assessment which is still currently used—the cardio–thoracic ratio (CTR). This is expressed as the transverse diameter of the cardiac silhouette by the addition of the greatest distances from the midline to the left and right cardiac borders, divided by the greatest horizontal distance between the inner borders of the ribs with the chest in the mid-inspiratory position [1,3,4]. The transverse cardiac diameter (TCD) may also be used as a parameter in the detection of cardiac enlargement or cardiomegaly; however, its use is limited in clinical practice. Different studies suggest various cut-off points for the CTR and TCD. Generally, cardiac enlargement is defined as a CTR > 0.5 and a TCD ≥ 155 mm in men and ≥145 mm in women in a posterior–anterior view [1,5]. The use of inaccurate CTR values may result in delayed or unwarranted diagnostics. Previous studies have shown that there is still a significant number of false-positive results, which contribute to misdiagnoses of cardiac enlargement [6]. Moreover, a normal cardiac silhouette does not always rule out cardiac enlargement due to, for example, the counterclockwise transverse rotation of the heart within the thorax.

Cardiac magnetic resonance (CMR) is a non-invasive imaging modality that can evaluate cardiac structures with great spatial resolution and tissue contrast [7]. The volumes and mass of the LV can be estimated with high accuracy and repeatability [7,8]. Therefore, CMR is considered to be the gold standard in their quantification [7]. However, this imaging modality requires specialist knowledge and is not always available. Echocardiography is a less expensive imaging modality used for the initial assessment of cardiac size and function; however, it too is not always readily available. Therefore, electrocardiography (ECG) and chest radiography, which are frequently performed as initial investigations, will not lose their relevance in daily clinical practice [1,9]. These two methods are widely used as screening tools for the investigation of cardiac chamber abnormalities or cardiac enlargement and may benefit from a new approach to improve their clinical performance [9,10].

The aim of this study was to assess the value of CTR and TCD and compare them with novel chest X-ray parameters in screening for cardiac enlargement. Importantly, this study focused on patients with cardiac enlargement determined using CMR, which is the gold standard for this assessment.

## 2. Materials and Methods

### 2.1. Study Group

We included 54 patients who had CMR and chest radiography performed between 2011 and 2015 in the Department of Diagnostic Imaging of the University Hospital, Kraków, Poland. Baseline data of patients were retrospectively obtained from available medical documentation. Our study was accepted by the local Ethics Committee.

### 2.2. Chest Radiography

**Figure 1:**
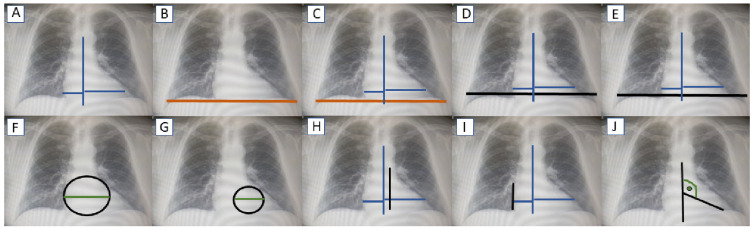
Schematic representation of the methodology of measurements of cardiac enlargement indicators and related measures: (**A**) transverse cardiac diameter, (**B**) transverse chest diameter, (**C**) cardiothoracic ratio 1, (**D**) cardiothoracic ratio 2, (**E**) cardiothoracic ratio 3, (**F**) basic spherical index, (**G**) left-side spherical index, (**H**) left diameter ratio, (**I**) right diameter ratio, (**J**) angle of inclination.

Chest radiographs were made in the posterior–anterior projection in the upright position with the chest in the mid-inspiratory state. The TCD was calculated as the maximal border of the cardiac silhouette, as previously described [10]. The CTR was evaluated with the use of a standard formula in which TCD was divided by the greatest horizontal distance between the inner borders of the chest. Moreover, the non-standard, additional formulas were used, in which TCD was divided by the horizontal distance between the inner boundaries of the ribs at the level of the right or left diaphragm. Additionally, five other new radiographic parameters (measured for each patient) were used for the assessment of changes in the cardiac silhouette due to cardiac enlargement on chest radiography in the posterior–anterior projection. These included basic spherical index (BSI), angle of inclination (IA), left diameter ratio (LDR), left-side spherical index (LSI), and right diameter ratio (RDR). Definitions of the parameters that were used are given in Table 1. The schematic methods of calculation for all indexes that were used are shown in Figure 1.

### 2.3. CMR Imaging

Images via CMR were made using a 1.5 T GE Signa HDxt scanner (General Electric, Milwaukee, WI, USA) as part of routine standard clinical protocols [8,11,12], as previously described [10,12,13]. Images were obtained using steady-state free-procession (SSFP) breath-hold cines. If indicated, a contrast agent was given and early and late gadolinium enhancement was assessed. LV parameters were measured using commercially available software (QMASS analysis). Cut-off values of LV mass (LVM) and LV end-diastolic volume (LVEDV) were obtained from a study performed by Petersen et al. (to date, this study is the largest to provide CMR-specific reference ranges for LV structure and function using SSFP sequences) [14]. Absolute LVEDV > 232 mL was considered abnormal in males, LVEDV > 175 mL was considered abnormal in females [14], and the indexed LVEDV was considered abnormal if >117 mL/m^2^ for men or 101 mL/m^2^ for women. Left ventricular hypertrophy (LVH) was defined as LVM > 148 g in men or >96 g in women or LVM/body surface area (BSA) > 72 g/m^2^ in men or >55 g/m^2^ in women [14]. Decreased LV ejection fraction (LVEF) was defined as LVEF < 50% [15].

### 2.4. Statistical Analysis

The Student’s *t*-test or Mann–Whitney U test was used to assess differences in continuous variables between two groups, as appropriate. Pearson’s or Spearman’s correlation was used to assess the degree of association between two continuous variables. Continuous variables were depicted as means ± standard deviations or medians and interquartile ranges (IQRs). The Pearson χ^2^ test was used for the assessment of associations between categorical variables. Categorical variables are shown as numbers and percentages. Analysis of receiver operating characteristics was used to determine the best variable or combination of variables to discriminate between patients with abnormally increased LVEDV, LV end-systolic volume (LVESV), LVM, or LVEF and the remaining patients. Statistical significance was defined as *p* < 0.05 for all tests. Moreover, specificity, sensitivity, positive predictive value, negative predictive value, accuracy, and likelihood ratio were calculated for CTR, TCD, and BSI. Statistical analyses were performed using IBM SPSS Statistics (version 24, IBM Corp., Armonk, NY, USA), while 95% confidence intervals (CI) were calculated using the MedCalc software (available at: https://www.medcalc.org).

## 3. Results

### 3.1. Study Group

In our study, we included 54 patients (16.7% female) with a median age of 41.5 (28.8–62.3) years. In the study group, arterial hypertension was observed in 22 patients (40.7%), heart failure in 34 patients (63.0%), coronary artery disease in 21 patients (38.9%), dyslipidemia in 20 patients (37.0%), and diabetes mellitus in 8 patients (14.8%), while smoking was reported in 13 patients (24.1%). Median LVEF was 42.5% (24.5–60.0), LVEDV was 189.8 mL (164.5–281.2), LVESV was 101.5 mL (66.4–206.0), and LVM was 159.6 g (127.2–203.7). LVEF was decreased in 32 patients (59.3.%), while other LV parameters were increased in 20 (37.0%), 27 (50.0%), and 39 (72.2%) patients, respectively.

### 3.2. Chest X-Ray Parameters as Predictors of Cardiac Enlargement

**Figure 2:**
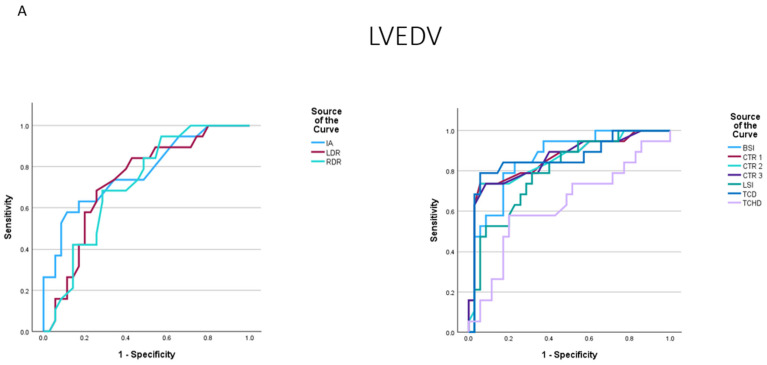

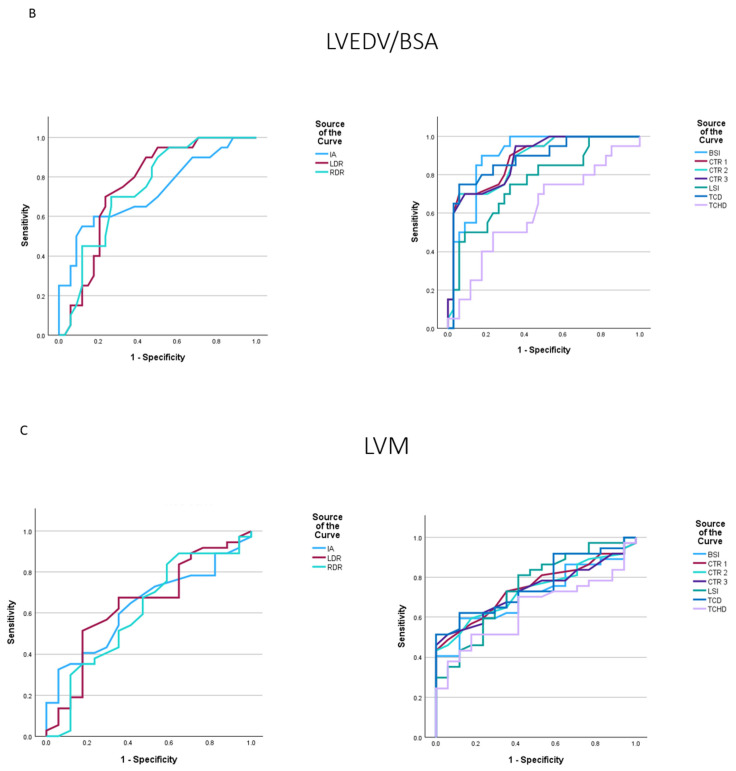

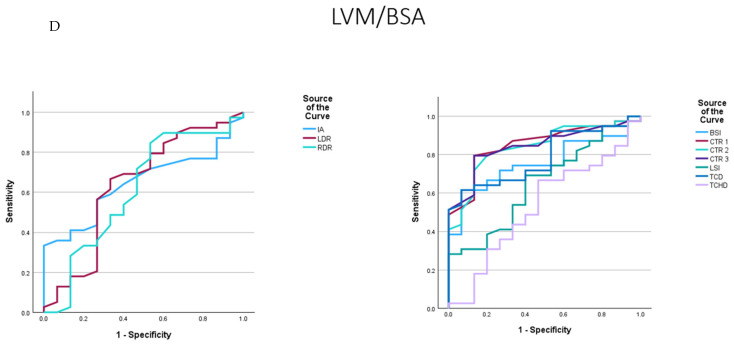
Area under the curve of radiographic measures for the prediction of increased left ventricular end-diastolic diameter (**A**,**B**) or left ventricular hypertrophy (**C**,**D**) determined by cardiac magnetic resonance. Abbreviations: BSA—body surface area, BSI—basic spherical index, CTR—cardio–thoracic ratio, IA—angle of inclination, LDR—left diameter ratio, LVEDV—left ventricular end-diastolic volume, LVM—left ventricular mass, LSI—left-side spherical index, RDR—right diameter ratio, TCD—transverse cardiac diameter, TCHD—transverse chest diameter.

Cardiac enlargement based on TCD, CTR 1, CTR 2, CTR 3, and BSI was observed in 29 (53.7%), 16 (29.6%), 18 (33.3%), 17 (31.5%), and 23 (42.6%) patients, respectively. The best predictors of both LVH and increased LVEDV diagnosed in CMR were TCD, CTR 1, CTR 2, CTR 3, and BSI (Table 2, Figure 2). When cardiac enlargement was defined as LVEDV > 232 mL in men or > 175 mL in women or LVEDV/BSA >117 mL/m^2^ in men or >101 mL/m^2^ in women, all chest X-ray parameters except transverse chest diameter (TCHD), as expected, were predictors of cardiac enlargement (Table 2). When LVH was defined as LVM > 148 g in men or >96 g in women, only TCD, CTR 1, CTR 2, CTR 3, BSI, and LSI were predictors of LVH. After indexing LVM to BSA and using the following cut-off points for LVH > 72 g/m^2^ in men or >55 g/m^2^ in women, TCD, CTR 1, CTR 2, CTR 3, and BSI were the only predictors of LVH (Table 2).

### 3.3. Correlations Between Chest X-Ray and LV Parameters Assessed by CMR

**Figure 3:**
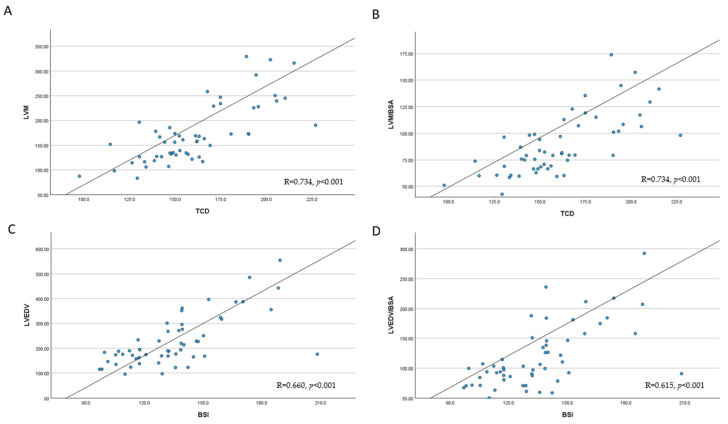
Correlations between selected chest radiographic and left ventricular parameters assessed by cardiac magnetic resonance imaging. (**A**) LVM and TCD, (**B**) LVM/BSA and TCD, (**C**) LVEDV and BSI, (**D**) LVEDV/BSA and BSI. For abbreviations, see Figure 2.

In our study, CTR 1, 2, and 3, TCD; BSI; and LSI showed significant positive correlations with LVM, LVEDV, and LVESV and negative correlations with LVEF (Table 3). LDR, RDR, and IA showed significant positive correlations with LVEF and negative correlations with LVM and LVESV. For LVM and LVM/BSA, the strongest positive correlations were observed with TCD (both R = 0.734, *p* < 0.001; Table 3), while, for LVEDV and LVEDV/BSA, the strongest positive correlations were observed with BSI (R = 0.660, *p* < 0.001 and R = 0.615, *p* < 0.001, respectively) (Figure 3).

### 3.4. Associations of Chest X-Ray Parameters and LV Abnormalities Diagnosed in CMR

When chest X-ray parameters were analyzed as categorical variables, we observed that all of them were more frequently positive in patients with CMR-LVH diagnosed based on non-indexed and indexed LVM when compared with the remaining patients (Table 4 and Table 5).

Similarly, we noted that all chest X-ray parameters were more frequently positive in patients with CMR-LVEDV enlargement diagnosed based on non-indexed and indexed LVEDV when compared with the remaining patients (Table 6 and Table 7). The McNemar test revealed that only TCD and TCD used together with BSI were in agreement with CMR-LVH defined both using non-indexed LVM and LVM indexed to BSA (Table 3 and Table 4). Conversely, only TCD and TCD used together with BSI were not in agreement with cardiac enlargement defined as LVEDV > 232 mL in men or >175 mL in women or LVEDV/BSA > 117 mL/m^2^ in men or >101 mL/m^2^ in women (Table 6 and Table 7).

### 3.5. Specificity and Sensitivity of Chest Radiographic Parameters

TCD, as a single parameter, had the best sensitivity in LVH prediction defined as LVM/BSA (66.7%, 95% CI: 49.8–80.9%). However, CTR 1, CTR 2, and CTR 3 had greater specificity than TCD, approaching 100% (Table 8).

A value of BSI > 135 (the cut-off point defined in the ROC analysis) demonstrated good sensitivity and specificity in the prediction of LVH using LVM/BSA (56.4%, 95% CI: 39.6–72.2% and 93.3%, 95% CI: 68.1–99.8%, respectively). The best sensitivity and good specificity were observed when TCD and BSI parameters were used together (69.2%, 95% CI: 52.4–83.0% and 80.0%, 95% CI: 51.9–95.7%, respectively). CTR 1, CTR 2, and CTR 3 had the best specificity in the prediction of cardiac enlargement, defined as LVEDV/BSA > 117 mL/m^2^ in men or >101 mL/m^2^ in women (Table 9).

When TCD and BSI parameters were used together in the prediction of cardiac enlargement, defined as LVEDV/BSA > 117 mL/m^2^ in men or >101 mL/m^2^ in women, we observed the greatest sensitivity (90%, 95% CI: 68.3–98.8%); however, specificity was the lowest when compared to other chest X-ray parameters (64.7%, 95% CI: 46.5–80.3%). In the prediction of cardiac enlargement, defined as LVEDV/BSA > 117 mL/m^2^ in men or >101 mL/m^2^ in women, BSI > 137.5 had the best values of sensitivity and specificity when considered as a screening tool (85.0%, 95% CI: 62.1–96.8% and 82.4%, 95% CI: 65.5–93.2%, respectively). A summary of the sensitivity and specificity results, categorized by performance is presented in Table 10.

## 4. Discussion

Chest radiographs are common radiologic examinations ordered in clinical practice and are indicated for initial examination in many diagnostic pathways and as a screening tool [16]. Chest radiography is a valuable component of the diagnostic process in heart failure patients and is also relevant in patients with hypertension [17,18,19]. In clinical practice, cardiac enlargement may be evaluated using CTR. Previous studies assessing cardiac enlargement in chest radiographs have focused mostly on CTR. In a recent meta-analysis consisting of five studies that included 371 adult patients, it was shown that CTR had 86.2% sensitivity, 25.2% specificity, 42.5% positive predictive value, and 74.0% negative predictive value for cardiac enlargement [6]. Our study showed that TCD and BSI had better sensitivity than CTR in the prediction of LVH and increased LVEDV. However, the specificity of CTR was the greatest when compared to other parameters. Some discrepancies between the above meta-analysis and our study may result from a different method of defining cardiac enlargement and draw attention to the need for appropriate indexation, not only of LVM but also of other LV parameters [13]. Additionally, the method of LV diameter evaluation (echocardiography vs. CMR) and the different sizes of the studied groups are not insignificant.

So far, there have only been weak correlations between CTR and LV volumes and LV dysfunction derived from radionuclide, echocardiographic, or CMR studies [20,21,22,23]. Limited studies investigating TCD have shown that correlations with LVEDV, LVESV, LVEF, and LVM were stronger for TCD when compared to CTR [1,24,25]. It has also been demonstrated that TCD may be a better indicator of LVH than CTR [10]. Similarly, our current study revealed that only TCD and TCD used together with BSI were in agreement with CMR-LVH when defined using both non-indexed LVM and LVM/BSA. Our study demonstrated that CTR 1, 2, and 3; TCD; BSI; and LSI showed moderately significant positive correlations with LVM, LVEDV, and LVESV and negative correlations with LVEF. The better usability of TCD may result from the fact that CTR is influenced by more factors than TCD [6,26,27]; however, TCD inherently captures only a single dimension of the complex three-dimensional structure of the heart [4,28,29]. This measurement predominantly reflects the diameters of the LV and the right atrium, as these chambers contribute significantly to the lateral extent of the cardiac silhouette in a posterior–anterior chest radiograph. However, TCD may also be influenced by the dilatation of other cardiac chambers, such as the right ventricle and the left atrium, which can expand and alter the contour of the cardiac silhouette. Additionally, pathologies of the aorta, such as aneurysmal dilatation, can further affect TCD measurements by displacing or enlarging parts of the cardiac outline. The CTR may be influenced by body posture, respiratory phases, or decreases in the transverse chest diameter in older age [6]. Elderly patients may have reduced bone mineral density and a “bell-shaped” costal deformity, which leads to a greater CTR [26]. Interestingly, in a study on 110 elderly women, it was demonstrated that CTR increased by 2.0% over 9 years of follow-up [30]. Similar findings were observed in a study investigating 243 men over 12 years of follow-up [31]. Moreover, patients with chronic obstructive pulmonary disease have larger lung volumes and relatively lower CTR [32]. Measurement errors are also very significant in inappropriate cardiac silhouette assessment. Measurement of the TCHD, which is the parameter used for the assessment of CTR, could represent a source of error in the evaluation of cardiac enlargement. Our study confirmed that different levels in which measurements of TCHD were made for CTR assessment may affect the clinical approach. Moreover, it should be noted that more precise diagnostics (but with increased dosage of radiation) could be provided using computed tomography, which may add information regarding intravascular or intracardiac structures as well as precise abnormalities observed in the lungs [33,34,35,36].

The prediction of increased LVEDV or LVH may also be performed using features other than the CTR and TCD [37]. Dislocations of the cardiac apex leftward and toward the diaphragm are seen with increased LVEDV or LVH. However, our study did not demonstrate the usefulness of the novel IA parameter, in which values depend on the cardiac apex displacement. Increased LVEDV and LVH also tends to enhance the convexity of the left cardiac border. Interestingly, our study suggests that out of the novel parameters assessing changes in cardiac silhouettes (BSI, LSI), BSI may be useful in the prediction of cardiac enlargement. In the case of LVH or increased LVEDV, the cardiac silhouette becomes wider rather than taller. However, novel parameters evaluating the ratio of width to height of the heart (LDR, RDR) did not show better suitability in the prediction of LVH or increased LVEDV when compared to other parameters. Importantly, in future clinical research and practice, chest radiographs could be used by algorithms based on artificial intelligence, potentially combined with biomarkers derived from peripheral blood or ECG monitoring [38,39,40,41,42,43].

Our study has several limitations that warrant discussion. First, the retrospective nature of this study introduced potential biases, including the timing of imaging studies. Chest radiographs and CMR examinations were not always performed on the same day, which may have resulted in changes in the clinical state of patients between the two imaging modalities, potentially affecting cardiac dimensions. Additionally, CTR and TCD do not take into account the cardiac cycle and changes in the size of cardiac chambers throughout the cardiac cycle. Second, the relatively small sample size limits the generalizability of the findings. While the results provide valuable insights, larger and more diverse cohorts are necessary to confirm the utility of the novel radiographic indexes across different populations and clinical scenarios, including the monitoring of cardiac enlargement. Third, measurement variability is an inherent limitation when assessing chest radiographic parameters. Factors such as patient positioning, respiratory phase, and radiographic technique can influence measurements of TCD and CTR, leading to potential inaccuracies [2,5,6]. Cardiac enlargement due to right ventricular or atrial enlargement was not evaluated, and conclusions regarding the specificity of TCD and BSI are therefore limited to cases of LV enlargement (LVH or increased LVEDV). Finally, the retrospective study design did not account for longitudinal changes in cardiac dimensions. Evaluating the performance of these indices in a prospective study with serial imaging would potentially better determine their utility in clinical practice for monitoring cardiac enlargement over time in selected cases. Despite these limitations, this study provides important preliminary evidence supporting the utility of TCD and BSI as radiographic indexes for the evaluation of cardiac enlargement while highlighting areas for future research and validation in larger population studies to confirm their applicability and generalizability.

## 5. Conclusions

A systematic approach to interpreting chest radiographs is recommended to enable a comprehensive assessment of cardiac structures. TCD may be useful in the assessment of patients suspected of having cardiac enlargement. In addition, CTR and BSI serve as complementary tools for a more precise approach. Finally, TCD appears particularly useful for the prediction of LVH, while BSI demonstrates greater utility as an indicator of increased LVEDV.

## Figures and Tables

**Table 1:** Indexes and related measures of cardiac enlargement based on chest radiographic parameters and their definitions.

Indexes	Definition
TCD	The transverse cardiac diameter was measured on the posterior–anterior film by drawing a vertical line through the vertebral bodies and calculating the sum of segments drawn perpendicular from the midline to the farthest edge of the cardiac silhouette in both directions.
TCHD	The greatest horizontal distance between the inner borders of the ribs with the chest in the mid-inspiratory position.
CTR 1	TCD/TCHD
CTR 2	TCD/horizontal distance between the inner borders of the ribs with the chest in the mid-inspiratory position at the level of the right diaphragm.
CTR 3	TCD/horizontal distance between the inner borders of the ribs with the chest in the mid-inspiratory position at the level of the left diaphragm.
BSI	The BSI represents the diameter of a theoretical circle to which both the left and right heart contours are tangent. It provides a metric for the assessment of changes in the cardiac silhouette that occur with LV enlargement. Clinically, the BSI is significant as it reflects not only the transverse diameter but also the overall shape of the heart, offering a more nuanced perspective than traditional measurements such as the CTR.
LSI	The LSI quantifies the diameter of the circle tangent to the left-most convex contour of the heart. This index focuses on the left heart border, which is more prominently affected in cases of LV enlargement, making it particularly relevant for evaluating asymmetric cardiac enlargement.
LDR	The LDR is calculated as the ratio of the distance between the lower wall of the left bronchus and the left diaphragm to the TCD. This index highlights structural changes in the lower left region of the cardiac silhouette, which often occur in LV dilatation.
RDR	The RDR evaluates the diameter of the line connecting the upper visible right heart contour to the right diaphragm, normalized to TCD. While primarily used for symmetry assessment, this parameter can help differentiate between right- and left-sided cardiac contributions to overall enlargement.
IA	The IA measures the angle formed by the largest oblique diameter of the heart toward the apex and a central horizontal line. This index reflects apex displacement due to left ventricular enlargement and helps to identify changes in the orientation of the cardiac silhouette.

Abbreviations: BSI—basic spherical index, CTR—cardiothoracic ratio, IA—angle of inclination, LDR—left diameter ratio, LSI—left-side spherical index, LV—left ventricular, RDR—right diameter ratio, TCD—transverse cardiac diameter, TCHD—transverse chest diameter.

**Table 2:** Area under the curve analyses of chest X-ray parameters in the prediction of left ventricular parameters determined by cardiac magnetic resonance.

Chest X-Ray Parameters	LVM > 148 g (M) or >96 g (F)	LVM / BSA > 72 g/m^2^ (M) or >55 g/m^2^ (F)	LVEDV > 232 mL (M) or >175 mL (F)	LVEDV/BSA > 117 mL/m^2^ (M) or >101 mL/m^2^ (F)
TCD	0.764 (0.002)	0.785 (0.001)	0.864 (<0.001)	0.880 (<0.001)
TCHD	0.637 (0.12)	0.530 (0.735)	0.629 (0.121)	0.610 (0.179)
CTR 1	0.764 (0.004)	0.853 (<0.001)	0.859 (<0.001)	0.882 (<0.001)
CTR 2	0.734 (0.006)	0.840 (<0.001)	0.855 (<0.001)	0.872 (<0.001)
CTR 3	0.736 (0.006)	0.844 (<0.001)	0.859 (<0.001)	0.879 (<0.001)
BSI	0.715 (0.01)	0.759 (0.003)	0.861 (<0.001)	0.896 (<0.001)
LSI	0.646 (0.004)	0.650 (0.12)	0.795 (<0.001)	0.757 (0.002)
LDR	0.644 (0.09)	0.637 (0.12)	0.731 (0.005)	0.759 (0.002)
RDR	0.607 (0.21)	0.612 (0.21)	0.716 (0.009)	0.743 (0.003)
IA	0.633 (0.12)	0.646 (0.1)	0.774 (0.001)	0.726 (0.006)

Data are presented as the area under the curve (*p*-value). Abbreviations: BSA—body surface area, F—female, LVEDV—left ventricular end-diastolic volume, LVM—left ventricular mass, M—male. For other abbreviations, see Table 1.

**Table 3:** Correlations between chest X-ray and left ventricular parameters assessed in cardiac magnetic resonance.

Chest X-Ray Parameters	LVEDV	LVEDV/BSA	LVM	LVM/BSA	LVEF	LVESV
TCD	0.626 (<0.001)	0.584 (<0.001)	0.734 (<0.001)	0.734 (<0.001)	−0.690 (<0.001)	0.676 (<0.001)
TCHD	0.354 (0.009)	0.174 (0.21)	0.590 (<0.001)	0.401 (0.003)	−0.230 (0.09)	0.353 (0.009)
CTR 1	0.519 (<0.001)	0.590 (<0.001)	0.516 (<0.001)	0.645 (<0.001)	−0.595 (<0.001)	0.565 (<0.001)
CTR 2	0.506 (<0.001)	0.567 (<0.001)	0.524 (<0.001)	0.641 (<0.001)	−0.570 (<0.001)	0.548 (<0.001)
CTR 3	0.517 (<0.001)	0.580 (<0.001)	0.510 (<0.001)	0.634 (<0.001)	−0.596 (<0.001)	0.572 (<0.001)
BSI	0.660 (<0.001)	0.615 (<0.001)	0.634 (<0.001)	0.644 (<0.001)	−0.690 (<0.001)	0.740 (<0.001)
LSI	0.552 (<0.001)	0.448 (0.001)	0.548 (<0.001)	0.452 (0.001)	−0.392 (0.003)	0.523 (<0.001)
LDR	−0.314 (0.02)	−0.262 (0.06)	−0.450 (0.001)	−0.461 (<0.001)	0.463 (<0.001)	−0.432 (0.001)
RDR	−0.255 (0.06)	−0.227 (0.1)	−0.349 (0.01)	−0.346 (0.01)	0.448 (<0.001)	−0.375 (0.005)
IA	−0.341 (0.012)	−0.329 (0.015)	−0.408 (0.002)	−0.409 (0.002)	0.459 (<0.001))	−0.434 (0.001)

Data are presented as R (*p*-value). Abbreviations: LVEF—left ventricular ejection fraction, LVESV—left ventricular end-systolic volume. For other abbreviations, see Table 1 and Table 2.

**Table 4:** Chest X-ray criteria for the prediction of left ventricular hypertrophy, based on non-indexed left ventricular mass.

Positive Chest X-Ray Criteria	LVM > 148 g (M) or > 96 g (F) (*n* = 37)		LVM ≤ 148 g (M) or ≤ 96 g (F) (*n* = 17)		McNemar Test	*p*-Value
	TP	FN	FP	TN		
CTR 1	16 (43.2%)	21 (56.8%)	0 (0.0%)	17 (100.0%)	<0.0001	0.001
CTR 2	17 (45.9%)	20 (54.1%)	1 (5.9%)	16 (94.1%)	<0.0001	0.004
CTR 3	17 (45.9%)	20 (54.1%)	0 (0.0%)	17 (100.0%)	<0.0001	0.001
TCD	24 (64.9%)	13 (35.1%)	5 (29.4%)	12 (70.6%)	0.1	0.02
BSI	21 (56.8%)	16 (43.2%)	2 (11.8%)	15 (88.2%)	0.001	0.002
TCD and BSI	24 (64.9%)	13 (35.1%)	6 (35.3%)	11 (64.7%)	0.17	0.04

Data are presented as numbers (percentages). Abbreviations: FN—false negative, FP—false positive, TN—true negative, TP—true positive. For other abbreviations, see Table 1 and Table 2.

**Table 5:** Chest X-ray criteria for the prediction of left ventricular hypertrophy based on indexed left ventricular mass and body mass index.

Positive Chest X-Ray Criteria	LVM / BSA > 72 g/m^2^ (M) or >55 g/m^2^ (F) (*n* = 39)		LVM / BSA ≤ 72 g/m^2^ (M) or ≤55 g/m^2^ (F) (*n* = 15)		McNemar Test	*p*-Value
	TP	FN	FP	TN		
CTR 1	16 (41.0%)	23 (59.0%)	0 (0.0%)	15 (100.0%)	<0.0001	0.003
CTR 2	17 (43.6%)	22 (56.4%)	1 (6.7%)	14 (93.3%)	<0.0001	0.01
CTR 3	17 (43.6%)	22 (56.4%)	0 (0.0%)	15 (100.0%)	<0.0001	0.002
TCD	26 (66.7%)	13 (33.3%)	3 (20.0%)	12 (80.0%)	0.02	0.002
BSI	22 (56.4%)	17 (43.6%)	1 (6.7%)	14 (93.3%)	<0.0001	0.001
TCD and BSI	27 (69.2%)	12 (30.8%)	3 (20.0%)	12 (80.0%)	0.04	0.001

Data are presented as numbers (percentages). For abbreviations, see Table 1 and Table 2.

**Table 6:** Chest X-ray criteria for the prediction of increased left ventricular end-diastolic diameter based on non-indexed left ventricular end-diastolic diameter.

Positive Chest X-Ray Criteria	LVEDV > 232 mL (M) or >175 mL (F) (*n* = 19)		LVEDV ≤ 232 mL (M) or ≤175 mL (F) (*n* = 35)		McNemar Test	*p*-Value
	TP	FN	FP	TN		
CTR 1	14 (73.7%)	5 (26.3%)	2 (5.7%)	33 (94.3%)	0.45	<0.0001
CTR 2	14 (73.7%)	5 (26.3%)	4 (11.4%)	31 (88.6%)	1	<0.0001
CTR 3	14 (73.7%)	5 (26.3%)	3 (8.6%)	32 (91.4%)	0.73	<0.0001
TCD	16 (84.2%)	3 (15.8%)	13 (37.1%)	22 (62.9%)	0.02	0.001
BSI	15 (78.9%)	4 (21.1%)	8 (22.9%)	27 (77.1%)	0.4	<0.0001
TCD and BSI	16 (84.2%)	3 (15.8%)	14(40.0%)	21 (60.0%)	0.01	0.002

Data are presented as numbers (percentages). For abbreviations, see Table 1 and Table 2.

**Table 7:** Chest X-ray criteria for the prediction of increased left ventricular end-diastolic diameter based on indexed left ventricular end-diastolic diameter and body mass index.

Positive Chest X-Ray Criteria	LVEDV/BSA > 117 mL/m^2^ (M) or >101 mL/m^2^ (F) (*n* = 20)		LVEDV/BSA ≤ 117 mL/m^2^ (M) or ≤101 mL/m^2^ (F) (*n* = 34)		McNemar Test	*p*-Value
	TP	FN	FP	TN		
CTR 1	14 (70.0%)	6 (30.0%)	2 (5.9%)	32 (94.1%)	0.29	<0.0001
CTR 2	14 (70.0%)	6 (30.0%)	4 (11.8%)	30 (88.2%)	0.75	<0.0001
CTR 3	14 (70.0%)	6 (30.0%)	3 (8.8%)	31 (91.2%)	0.51	<0.0001
TCD	17 (85.0%)	3 (15.0%)	12 (35.3%)	22 (64.7%)	0.04	<0.0001
BSI	17 (85.0%)	3 (15.0%)	6 (17.6%)	28 (82.4%)	0.51	<0.0001
TCD and BSI	18 (90.0%)	2 (10.0%)	12 (35.3%)	22 (64.7%)	0.01	<0.0001

Data are presented as numbers (percentages). For abbreviations, see Table 1 and Table 2.

**Table 8:** Sensitivity, specificity, PPV, PNV, accuracy, PLR, and NLR for chest X-ray parameters in the prediction of left ventricular hypertrophy defined as LVM / BSA > 72 g/m^2^ (M) or > 55 g/m^2^ (F).

Chest X-Ray Parameters	Sensitivity, %	Specificity, %	PPV, %	PNV, %	Accuracy, %	PLR	NLR
CTR 1	41.0 (25.6–57.9)	100.0 (78.2–100.0)	100.0 *	39.5 (33.4–45.9)	57.4 (43.2–70.8)	*	0.6 (0.5–0.8)
CTR 2	43.6 (27.8–60.4)	93.3 (68.1–99.8)	94.4 (71.2–99.2)	38.9 (31.9–46.4)	57.4 (43.2–70.8)	6.5 (1.0–44.9)	0.6 (0.4–0.8)
CTR 3	43.6 (27.8–60.4)	100.0 (78.2–100.0)	100.0 *	40.5 (34.1–47.3)	59.3 (45.0–72.4)	*	0.6 (0.4–0.7)
TCD	66.7 (49.8–80.9)	80.0 (51.9–95.7)	89.7 (75.5–96.1)	48.0 (35.6–60.6)	70.4 (56.4–82.0)	3.3 (1.2–9.4)	0.4 (0.3–0.7)
BSI > 137.5	56.4 (39.6–72.2)	93.3 (68.1–99.8)	95.7 (76.5–99.3)	45.2 (36.0–54.7)	66.7 (52.5–78.9)	8.5 (1.3–57.3)	0.5 (0.3–0.7)
TCD and BSI	69.2 (52.4–83.0)	80.0 (51.9–95.7)	90.0 (76.2–96.2)	50.0 (37.0–63.1)	3.5 (1.2–9.7)	0.4 (0.2–0.7)	0.4 (0.3–0.7)

Abbreviations: PPV—predictive positive value, PNV—predictive negative value, PLR—positive likelihood ratio, NLR—negative likelihood ratio. For other abbreviations, see Table 1. (*)—95% CI or PLR not available.

**Table 9:** Sensitivity, specificity, PPV, PNV, accuracy, PLR, and NLR for chest X-ray parameters in the prediction of cardiac enlargement defined as LVEDV/BSA > 117 mL/m^2^ (M) or >101 mL/m^2^ (F).

Chest X-Ray Parameters	Sensitivity, %	Specificity, %	PPV, %	PNV, %	Accuracy, %	PLR	NLR
CTR 1	70.0 (45.7–88.1)	94.1 (80.3–99.3)	87.5 (63.9–96.5)	84.2 (73.1–91.3)	85.2 (72.9–93.4)	11.9 (3.0–47.1)	0.3 (0.2–0.6)
CTR 2	70.0 (45.7–88.1)	88.2 (72.6–96.7)	77.8 (57.2–90.2)	83.3 (71.7–90.8)	81.5 (68.6–90.8)	6.0 (2.3–15.6)	0.3 (0.2–0.7)
CTR 3	70.0 (45.7–88.1)	91.2 (76.3–98.1)	82.4 (60.4–93.5)	83.4 (72.4–91.1)	83.3 (70.7–92.1)	7.9 (2.6–24.3)	0.3 (0.2–0.7)
TCD	85.0 (62.1–96.8)	64.7 (46.5–80.3)	58.6 (46.4–69.8)	88.0 (71.5–95.5)	72.2 (58.4–83.5)	2.4 (1.5–3.9)	0.2 (0.1–0.7)
BSI > 137.5	85.0 (62.1–96.8)	82.4 (65.5–93.2)	73.9 (57.3–85.7)	90.3 (76.5–96.4)	83.3 (70.7–92.1)	4.8 (2.3–10.2)	0.2 (0.1–05)
TCD and BSI	90.0 (68.3–98.8)	64.7 (46.5–80.3)	60.0 (48.2–70.8)	91.7 (74.3–97.7)	74.1 (60.4–85.0)	2.6 (1.6–4.1)	0.2 (0.04–0.6)

For abbreviations, see Table 1 and Table 8. Values are presented as numbers (percentages) or percentages (95% CI) or numbers. Calculations were made for 38 patients.

**Table 10:** Summary of sensitivity and specificity results for selected chest X-ray parameters, categorized by performance (high: ≥80%, moderate: 50–79%, low: <50%).

Metric for LVM/BSA	High (≥80%)	Moderate (50–79%)	Low (<50%)
Sensitivity	TCD, TCD + BSI	BSI, CTR 2, CTR 3	CTR 1
Specificity	CTR 1, CTR 2, CTR 3, BSI	TCD, TCD + BSI	None
**Metric for LVEDV/BSA**	**High (≥80%)**	**Moderate (50–79%)**	**Low (<50%)**
Sensitivity	TCD, BSI, TCD + BSI	CTR 1, CTR 2, CTR 3	None
Specificity	CTR 1, CTR 2, CTR 3, BSI	TCD, TCD + BSI	None

For abbreviations, see Table 1 and Table 2.

## Data Availability

The data underlying this article will be shared upon reasonable request to the first author of this paper.
